# A new method for urine electrofiltration and long term power enhancement using surface modified anodes with activated carbon in ceramic microbial fuel cells

**DOI:** 10.1016/j.electacta.2020.136388

**Published:** 2020-09-01

**Authors:** Iwona Gajda, Jiseon You, Carlo Santoro, John Greenman, Ioannis A. Ieropoulos

**Affiliations:** a Bristol BioEnergy Centre, Bristol Robotics Laboratory, University of the West of England, BS16 1QY, UK; b Biological, Biomedical and Analytical Sciences, University of the West of England, BS16 1QY, UK

**Keywords:** Microbial fuel cell, Activated carbon, Micro-nanostructure, Ceramic membrane, Catholyte production, Urine

## Abstract

This work is presenting for the first time the use of inexpensive and efficient anode material for boosting power production, as well as improving electrofiltration of human urine in tubular microbial fuel cells (MFCs). The MFCs were constructed using unglazed ceramic clay functioning as the membrane and chassis. The study is looking into effective anodic surface modification by applying activated carbon micro-nanostructure onto carbon fibres that allows electrode packing without excessive enlargement of the electrode. The surface treatment of the carbon veil matrix resulted in 3.7 mW (52.9 W m^−3^ and 1626 mW m^−2^) of power generated and almost a 10-fold increase in the anodic current due to the doping as well as long-term stability in one year of continuous operation. The higher power output resulted in the synthesis of clear catholyte, thereby i) avoiding cathode fouling and contributing to the active splitting of both pH and ions and ii) transforming urine into a purified catholyte - 30% salt reduction - by electroosmotic drag, whilst generating - rather than consuming – electricity, and in a way demonstrating electrofiltration. For the purpose of future technology implementation , the importance of simultaneous increase in power generation, long-term stability over 1 year and efficient urine cleaning by using low-cost materials, is very promising and helps the technology enter the wider market.

## Highlights

• Activated carbon modified anodes outperformed unmodified anodes.
• Activated carbon modification increased the specific surface area.
• Incisions in modified and unmodified anodes showed increase in power generation.
• Good strategy for anode packing in miniaturised MFCs and long term stability.
• MFCs with modified anodes generated clear catholyte from urine through electrofiltration.

## Graphical abstract

**Figure:**
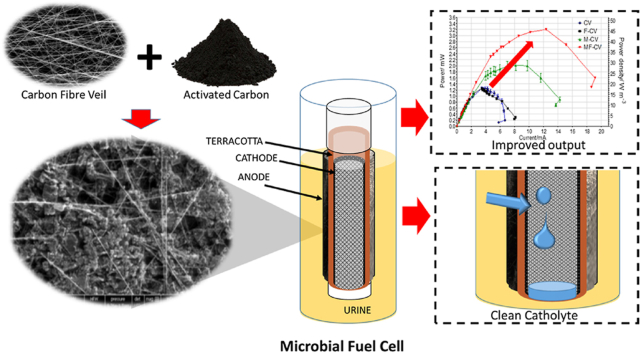

## 1 Introduction

Microbial Fuel Cells (MFCs) are transforming chemical energy locked in organic waste materials into electrical energy using natural bacterial metabolism [1]. Bacterial consortia form biofilms on the electrode surface (the anode), which functions as the ‘live engine’ of biomass utilisation into electric current with the additional benefit of waste treatment [2,3]. One of the substrates that has recently received significant attention is neat human urine, which has been reported as an ideal feedstock for energy generation from individual as well as collective MFC systems [6]. The ability of these electroactive organisms is bringing great opportunities for MFC technology scale up and implementation into real world applications, such as wastewater treatment, and it has the potential to make the overall process energy efficient [4]. In order to reach this goal, the system needs to be optimised in terms of the power performance as well as scale, cost and robustness [5]. For this purpose, unglazed ceramic separators have more recently been successfully implemented into MFC reactors [7,8] scaled-up stack systems [9] for field trials and practical applications [10]. This includes scaled-up prototypes as off-grid power systems fed with urine to power LED lights [11]. At the same time nutrients and water can be transported to the previously empty air breathing cathode compartment both by passive transport (osmosis) and active flux as a function of produced current [12]. In order to increase the power density, individual MFC reactors need to be scaled down to decrease ohmic losses and in turn multiplied in modules [10,13]. In this sense, the compact, modular systems are more power-efficient and have higher substrate removal rates [9]. The anode electrode is an essential component of the MFC setup as it provides support for bacterial biofilm and acts as a conduit for electrons released from substrate metabolism [14]. Suitable anode materials should possess high specific surface area, good electrical conductivity and be biocompatible as well as resistant to corrosion [14,15]; there is a vast selection of carbonaceous materials is being used for the anode construction [16]. The most common materials include carbon cloth [17], felt [18] and brush [19] as well as granular forms of carbon such as graphite [20] and activated carbon (GAC) [21]. In general, three-dimensional structures that increase the biotic/abiotic interface and possess higher anode surface area to reactor volume ratios are considered to be more efficient [22,23]. While the increase of the anode electrode surface area enhances the power output of the MFC itself [24], larger anodes, apart from being more costly, require more space in the anode chamber, thus constraining the design of more compact reactors [19] and are inherently more electrically resistive. Therefore, anodes with micro and nanoscale structures have been developed over the last decade, modified with carbon nanotubes [25], carbon black particles [26] and graphene [27,28], which promote electricity generation in MFCs thanks to increased specific surface area, improved conductivity and enhanced biofilm formation. Nanoscale would facilitate electron transfer by providing multiplexed and highly conductive pathways, whilst the microscale would support the microbial attachment. Such a strategy is an important factor in building efficient systems, however in order to implement the technology into larger scale, cost and fabrication simplicity should also be considered. Electrode designs are the greatest challenge in MFC manufacturing [29], therefore one of the readily available and economical materials has been carbon fibre veil due to its conformity into 3D structures [12]. Cost effective carbon veil has been shown to be efficient in lab-scale systems [13] and validated in larger-scale applications [11].

One of the readily available microporous types of carbon is activated charcoal powder which has a high surface area (800–1000 m^2^ g^−1^), and has also been widely used as an affordable and durable cathode catalyst [[30], [31], [32]]. Apart from the porous structure, the activated carbon exhibits a complex surface chemistry [33] that greatly benefits electrochemical energy storage [34] due to its supercapacitive as well as adsorption properties, which are widely used in advanced wastewater treatment and filtration [35]. As the ceramic cylinder-based MFCs exhibit another important feature of catholyte synthesis within the previously empty cathode chamber due to osmotic and electroosmotic forces, the power output (or amount of charge) directly correlates with ion transfer [12]. Another factor influencing the quality of the synthesised catholyte is also affected by the oxygen reduction reaction (ORR) resulting in the production of hydroxide ions elevating the pH and in effect the production of a caustic permeate.

The main purpose of this study was to investigate the feasibility of exploiting activated carbon nanoparticles coated onto the network of carbon fibre veil. This is a novel, cost effective and very simple preparation method using carbon fibre veil and a commercially available, edible activated carbon powder obtained from pyrolised coconut shells. The aim was to increase the anode electrode packing without the extensive increase of the anodic total macroscopic surface area and the total bulk (geometric) volume of the electrode. This allowed fitting into the miniaturised MFCs, which then formed stacks for increased efficiency and useable power levels. The purification is conducted whilst electricity is produced, which in turn initiates the electrofiltration and combines the electrokinetic transport of ions and movement of water molecules through electro-osmosis from the anode to the cathode. The electrochemical activity of the modified electrode, the morphology of the anode, and the overall MFC performance were therefore investigated. Scanning electron microscopy (SEM) was used to study the macro and microstructure of the modified anode. Two operation modes, i.e., batch mode feeding regime and continuous flow were employed. The physico-chemical properties and volume of the synthesised catholyte were used to characterise the electro-osmotic transfer process in this long term MFC study.

## 2 Materials and methods

### 2.1 MFC configuration and mode of operation

Single-chamber air-breathing cathode microbial fuel cells were used, where the ceramic structure acted as the separator between the anode and the cathode. Ceramic cylinders were handmade in house using terracotta clay (Bath Potters, UK) which was readily rolled to obtain uniform thickness, using a mechanised twin-roller (instrument for making home-made pasta), moulded around a tube and cut to size, air dried and kilned at 1070 °C for 10 min. The final product was 7 ± 0.2 cm tall with a wall thickness of 0.2 cm and the inner diameter of 1.5 cm. Porosity of the ceramic membrane was measured by the water absorption method and was found to be 11.7 ± 0.3%.

Activated carbon powder used for both anode modifications and assembly of all cathode electrodes was obtained from G Baldwin & Co health store as a food grade product and licensed pharmaceutical. It originated from coconut shells of the typical surface area of 1 g between 800 and 1000 m^2^ and particle size of 100 μm according to the specifications provided by supplier.

**Fig. 1:**
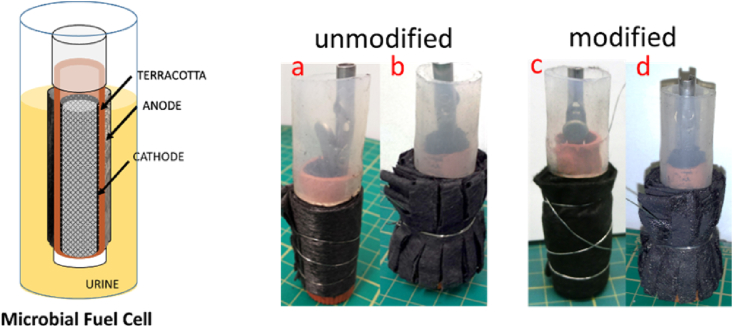
Microbial Fuel Cell scheme and assembled MFCs with tested anodes: a) CV unmodified and wrapped carbon veil, b) F-CV unmodified and incised carbon veil, c) M-CV-modified with activated carbon and wrapped carbon veil and d) MF-CV modified and incised carbon veil.

The cathode electrode was fabricated by pressing activated carbon (AC) paste on a polytetrafluoroethylene (PTFE) treated carbon veil used as current collector as previously described [36]. The air-breathing cathode (6.5 × 3.5 cm) was placed inside the cylinder where the AC paste was in direct contact with the ceramic surface. Stainless steel crocodile clip was used to connect to the cathode, whereas silicon tubing was attached to the top of the ceramic cylinder to prevent anolyte overflow into the inner cathode (Fig. 1). Cylinders were assembled with the use of plastic stoppers to close the bottom ends and sealed with the use of non-toxic sealant (Wet Water Sticky Stuff, UK) in order to isolate the outer anode and inner cathode chambers.

### 2.2 Anode material preparation

All anodes were prepared using identical 30 cm × 20 cm cut pieces of 20 g m^−2^ carbon fibre veil (PRF Composites, UK) as the main substratum.

**CV (Control)**- carbon veil was folded and wrapped around the cylinder, forming layers of the material, then it was tightened with stainless steel wire to hold the electrode in place and to make an electrical connection for the external circuit (Fig. 1A).

**F-CV**- carbon veil was wrapped the same way as in control group (CV) and secured with stainless steel wire in the middle, afterwards carbon fibre veil was cut towards a middle making 4 mm gaps between incisions but preserving the continuity of the carbon fibre to maintain electrical current collection. A “fluffy” or “brush-like” carbon veil structure is aimed to increase the electrode volume, opening available space for the biofilm formation (Fig. 1B).

**M-CV**- carbon veil was coated with activated carbon ink. The ink was prepared by blending 40 g of edible activated carbon purchased from health store (G Baldwins and Co., UK) with 5% PTFE (60% water dispersion in H_2_O, Sigma Aldrich) and 300 mL of deionised water. The blend was mixed for 2 min in order to obtain slurry and applied onto both sides of carbon veil. The sheets of such prepared material was then heat treated at 250 °C for 30 min. The activated carbon loading was 5 mg cm^−2^ (Fig. 1C).

**MF-CV**- it was assembled using modified carbon veil with activated carbon as in M-CV with the same incisions made as in F-CV (Fig. 1D).

Twelve MFC reactors as in triplicates of the four test groups, were inoculated using anaerobic sludge collected from Wessex Water treatment plant (Saltford, UK), which was mixed with hydrolysed (24 h old, pH 9.1 ± 0.1) human urine in the ratio of 1:1. The anolyte volume of each MFC was 70 ml. Subsequently, periodic feeding was maintained daily with batch replenishment of 30 mL of human urine, which was collected from healthy volunteers and pooled together. After 6 months, a continuous feeding of 100 % urine was set up to supply 242 mL/day per MFC using a 16-channel peristaltic pump (205 U, Watson Marlow, UK). The cathodes and ceramics used in this study were kept the same in all experimental groups.

### 2.3 Analysis and calculation

The output voltage was measured as voltage drop across an external resistor using a multi-channel Agilent 34972A data acquisition system connected to a PC. Polarisation curves were obtained by plotting voltage against current. The power generated was obtained by multiplying voltage and current and the power curves were plotted against current. Electrode potential was measured at variable resistance points during stabilised fuel cell operation (3 min intervals) from 30 kΩ to 3Ω. The different anode materials were electrochemically characterised using linear sweep voltammetry (LSV). LSVs were performed using SP-50 potentiostat (Bio-Logic), in a three-electrode configuration consisting of: Ag/AgCl 3 M (KCl) as reference electrode (inserted into the anode chamber close to the electrode), the tested anode as working electrode, and the MFC cathode as the counter electrode. LSVs were performed at a scan rate of 0.25 mV s^−1^. The cathode electrodes were also studied using LSV. In this case, the working electrode was the cathode, the counter electrode was the anode and the reference electrode was Ag/AgCl 3 M (KCl).

Surface morphology imagery of anode materials was recorded by scanning electron microscopy (SEM). Images were captured using a Philips XL30 scanning electron microscope (SEM). Samples were further prepared for microscopy by sputter coating in gold using an Emscope SC500. The quality of electrofiltration in newly formed permeate (catholyte) was assessed using electrical conductivity. Solution pH was measured using a handheld Hanna 8424 pH meter (Hanna, UK) and conductivity was measured with a 470 Jenway conductivity meter (Camlab, UK).

## 3 Results and discussion

### 3.1 Anode electrode morphology

**Fig. 2:**
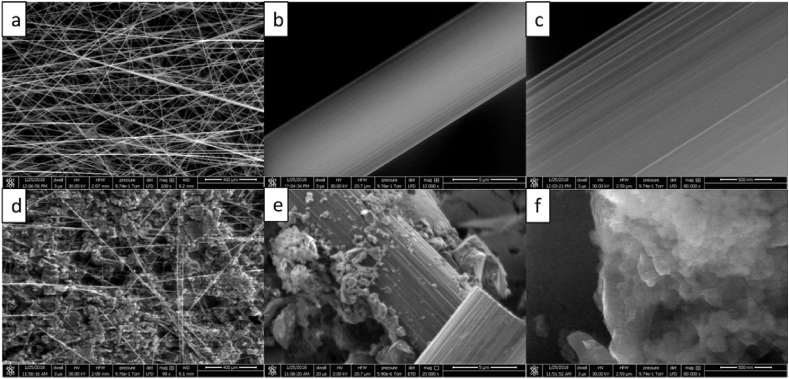
Scanning Electron Microscopy observing CV at (a) 100 × (b) 10,000 × (c) 80,000 × and M − CV (modified electrode with activated carbon) at (d) 99 × (e) 20,000 × (f) 80,000 ×.

Fig. 2 shows SEM images of the carbon fibre veil with and without the modification. It shows smooth long fibres forming a network with large void spaces between them where each fibre has cylinder-like shape (Fig. 2A and B). At higher magnification, shallow grooves along the long axis can be identified (Fig. 2C). By introducing the activated carbon modification (Fig. 2D), the gaps between fibres interconnect by an additional conductive material that forms clusters allowing electrical continuity and reducing the ohmic losses. Under high magnifications, it can be seen that the activated carbon clusters form a micro and nanoporous structure enhancing the surface roughness (Fig. 2E and F) that is visibly different from the smooth and uniform surface of an unmodified CV fibre (Fig. 2C). The combination of macro, micro and nano pores forms a rough surface that should provide more contact area for each single electroactive microorganism compared to the smooth carbon fibres. In previous studies, the effect of surface topology on bacterial attachment was predominant in macro-pores and grooves providing a preferential site for bacterial adhesion on the electrode surface [[23], [37]], while the actual microbe-electrode interaction was determined by the microscopic roughness [37].

### 3.2 MFC performance

**Fig. 3:**
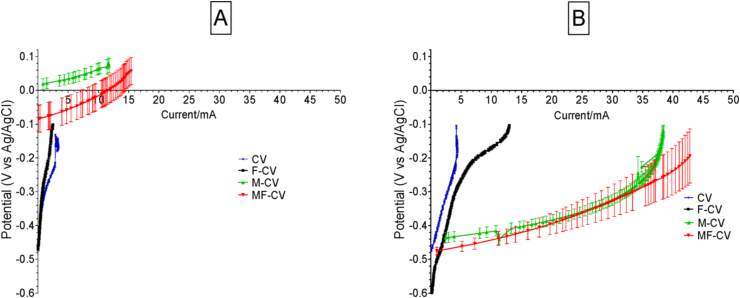
Linear Sweep Voltammetry (LSV) of the anodes during the start of the experiment (A) and after maturation (B).

To study the electrochemical activity of all tested anodes during biofilm maturation, linear sweep voltammetry (LSV) tests were conducted at the beginning of the experiment (Day 3, Fig. 3A) and after 10 days of MFC operation (Fig. 3B). Data show that during start-up, the unmodified anodes reached an open circuit potential (OCP) of −456 ± 4 mV (vs Ag/AgCl) for CV anodes and −467 ± 8 mV (vs Ag/AgCl) for the F-CV anodes however, the current obtained during the sweep was reaching 3.4 mA at −150 mV (vs Ag/AgCl) from CV and 2.1 mA from F-CV (Fig. 3A). OCP values of activated carbon modified electrodes showed much higher potential levels measuring +16 ± 23 mV (vs Ag/AgCl) for M-CV and −80 ± 40 mV (vs Ag/AgCl) for MF-CV. This indicated that the modified anode materials did not establish anaerobic conditions in the first three days of operation. This might be due to the hydrophobic nature of the applied mixture of activated carbon containing 5% of PTFE where the PTFE is used as a binder fixing the AC to the CV matrix and as a result that might have decreased the initial bacterial attachment on the electrode. It was shown before that an increase in PTFE content in commercially available carbonaceous anodes was increasing the start-up time of MFCs [38]. However, after 10 days of MFC maturing under external load, the anode LSV showed a shift of the OCP to more negative values (Fig. 3B) as M-CV reached −444 ± 15 mV (vs Ag/AgCl) and MF-CV achieved −477 ± 15 mV (vs Ag/AgCl) while the unmodified carbon veil (CV) showed −474 ± 5 mV (vs Ag/AgCl) and F-CV -609 ± 5 mV (vs Ag/AgCl). The current levels recorded at −200 mV (vs Ag/AgCl) were significantly different, depending on the anode used. The increasing trend current levels in Fig. 3b were found to be: CV producing 4.30 mA (189 μA cm^−2^) < F-CV with 7.21 mA (316 μA cm^−2^) < M-CV 37.05 mA (1628 μA cm^−2^) < MF-CV 42.8 mA (1881 μA cm^−2^), normalised by projected area of the cathode. Therefore the modification in M-CV and MF-CV showed almost a 10-fold increase in the anodic current in comparison to control, which was unmodified, wrapped CV.

**Fig. 4:**
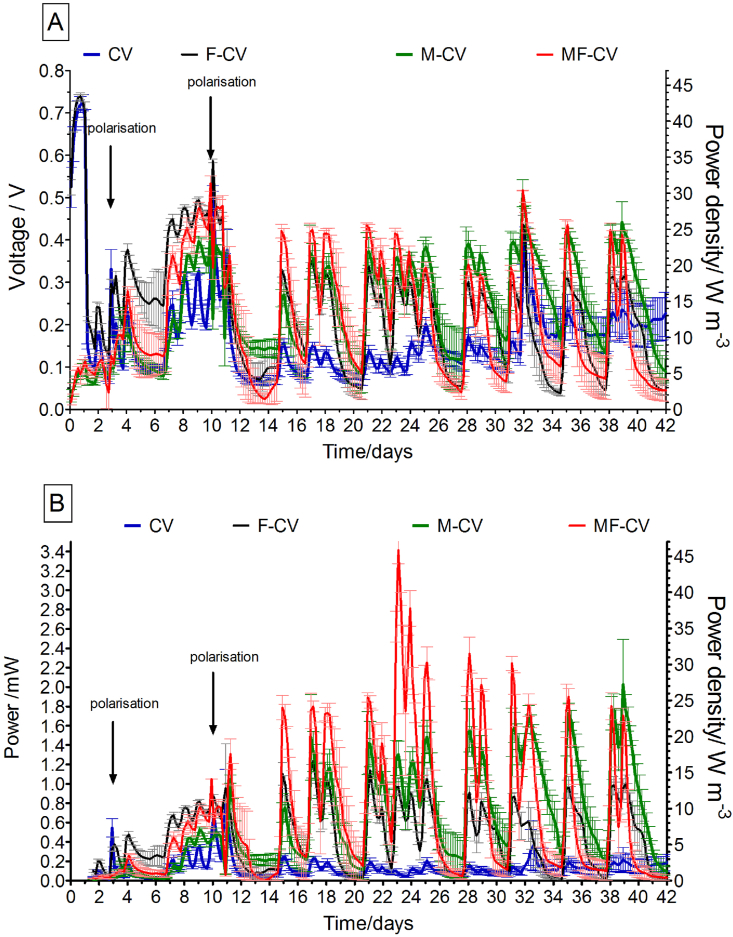
A) Voltage during inoculation showing open circuit voltage and under external load, B) Power output of all tested groups (in triplicate).

The initial performance of the MFCs observed 24 h after inoculation (Fig. 4 A) is indicating a slow start-up of the modified anodes (M-CV and MF-CV) when operated under open circuit voltage (OCV) that reached 100 mV while the unmodified anodes (CV and F-CV) reached levels of 730 mV. When the external load was connected (Fig. 4), the voltage of all MFCs initially dropped and showed a gradual increase of the output after each feedstock addition, then on day 10 reached similar levels as the unmodified MFCs. When the resistance value was lowered (from 300Ω to 100Ω), a significant difference between the anode types started to show. Fig. 4B presents the power performance from the moment MFCs were connected to the external load and shows that the peak power output was 3.4 ± 0.2 mW for the MF-CV and 2.0 ± 0.4 mW for the M-CV, while the anodes without modification reached up to 1.2 ± 0.1 mW for F-CV and 0.8 ± 0.4 mW for the CV. This suggests that the doping with activated carbon improved power performance. Anodic structural design (wrapped or incised) also played a role in power production indicating that incised and less convoluted anode is performing better in both modified and unmodified anode types. Both incised anode types also matured quicker reaching their optimum performance in shorter time.

### 3.3 Polarisation experiments

**Fig. 5:**
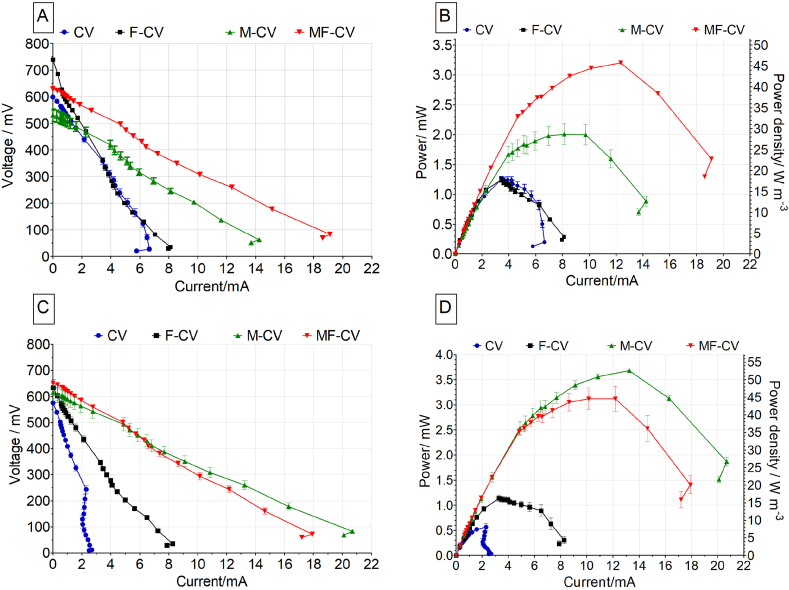
Polarisation curve performed on day 11 (A) and 32 (C). Power and power density curves of all tested MFCs on days 11 (B) and 32 (D).

During polarisation experiment performed on day 11, the OCV values were highest for the F-CV reaching 739 ± 7 mV, while CV reached 599 ± 1 mV, M-CV 530 ± 32 mV and MF-CV 628 ± 5 mV (Fig. 5A). The short circuit current (calculated at a cell voltage of approximately 70 mV) showed that modified anodes significantly outperformed the unmodified carbon veil materials with a maximum current output of 19.1 ± 0.1 mA for MF-CV 14.2 ± 0.9 mA from M-CV, while F-CV produced 7.0 ± 0.2 mA and CV 6.5 ± 0.4 mA. Power curves illustrated in Fig. 5B show that the maximum power reached by the MF-CV was 3.2 ± 0.1 mW and 2.0 ± 0.2 mW for the M-CV while both CV and F-CV reached 1.2 ± 0.1 mW. While the MFCs were operated under batch mode, the maturation was still continuing as can be seen in Fig. 4B. On day 32, the polarisation test was repeated and it was observed that the OCV value reached by the CV was 575 ± 5 mV and 634 ± 45 mV for F-CV while 615 ± 35 mV and 651 ± 4 mV for M-CV and MF-CV, respectively. Power performance in Fig. 5D shows that the maximum power reached by the M-CV was 3.7 ± 0.1 mW (52.9 Wm^-3^ and 1626 mWm^−2^) and 3.1 ± 0.2 mW (44.3 Wm^-3^ and 1362 mWm^−2^) for the MF-CV while for the unmodified anode types, F-CV reached 1.1 ± 0.1 mW (15.7 Wm^-3^ and 483 mWm^−2^) and CV reached 0.6 ± 0.1 (8.3 Wm^-3^ 263 mWm^−2^) mW. Power densities calculated with regard to the total anode or the total cathode area in Table 1 suggest that the power output of the modified anodes M-CV and MF-CV in this study are similar to previously reported values for microporous modification of carbon veil anode with carbon black particles [26], which reported 60.7 mW m^−2^. To the best of the Authors’ knowledge, this power output is the highest recorded so far from any ceramic MFC operated with human urine.

**Table 1:** Maximum absolute (raw) power from each MFC in this study and power densities calculated with respect to the total anode and total cathode macro-surface area.

MFC tested	Max. Absolute power (mW)	Max. Power density total anode area (mWm^−2^)	Max. Power density total cathode area (mWm^−2^)
CV	1.2	20.0	527.5
F-CV	1.2	20.0	527.5
M-CV	3.7	61.7	1626.4
MF-CV	3.1	51.7	1362.6

### 3.4 Long term performance

**Fig. 6:**
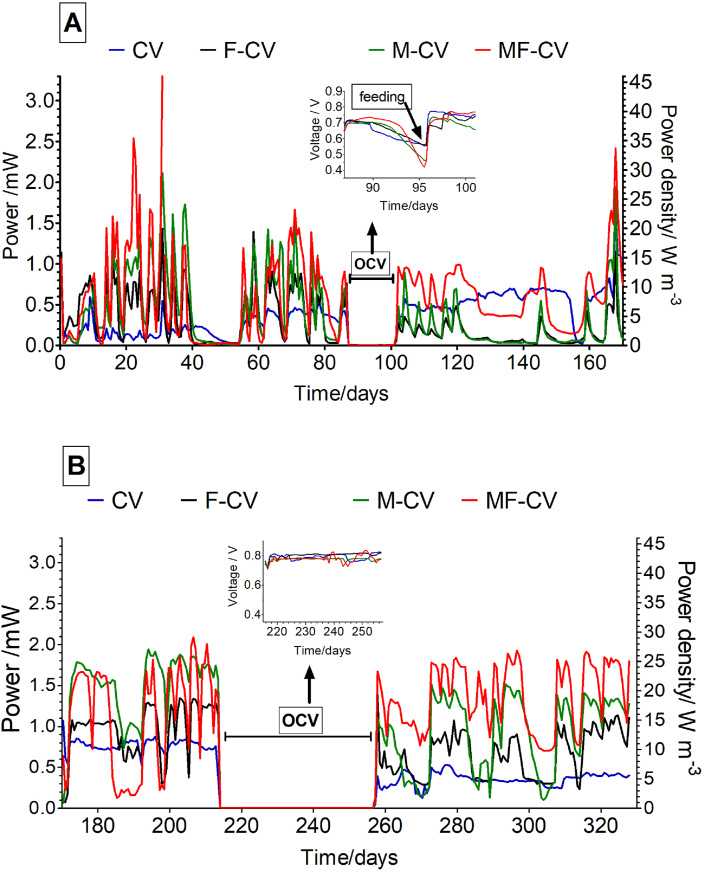
Long term behaviour of the tested MFCs over 1 year. The output shows mean value of triplicates in each group where A) MFCs are operated under batch-mode during first 170 days and B) MFCs are connected to the continuous mode feeding regime for the next 6 months.

For the purpose of investigating the MFC suitability for the real world implementations, it is important to study the system endurance in long-term operation. Fig. 6 shows MFC operation over the 12 month period where initially batch feeding was employed for the 170 days and subsequently continuous flow was operated for the rest 195 days. Even though continuous flow was implemented, a periodic decrease in output was observed and this is due to the cathode flooding where the catholyte formed inside the cylinder filled up the whole of the cathode chamber covering the cathode material above the connection point and above the anolyte level. Each removal of formed catholyte resulted in power being restored. The flooding is due to the electro-osmotic drag where current induced electrokinetic transport of water results in catholyte accumulation [12,39]. This is also supported by Fig. 6b (inset OCV) where cathode flooding did not occur.

**Fig. 7:**
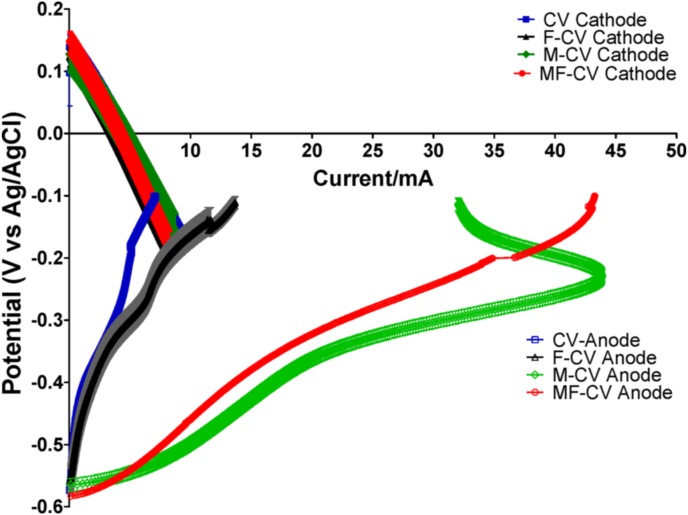
LSV performed after 8 months of MFC operation showing both anode and cathode LSV curves.

The conductive carbonaceous network is able to harvest the electrons from the attached microorganisms that strongly enhances the anodic performance even after long-term operation (Fig. 7), which underlines the robustness of the modifications in terms of adhesion stability of activated carbon particles onto the carbon veil matrix. In fact, anode polarisation curves showed that the modification with activated carbon enhanced the current generated (at identical potential values). Lower slopes of the modified anode can also be attributed to lower ohmic resistance of the anode electrode. Cathode polarisation curves were very similar and could be almost overlapped (Fig. 7). This demonstrated that the differences identified in the overall polarisation curves were given by the difference in the anode behaviour. Scaling up MFC size reduces electric current production which is linked to volume-based resistance [40,41], as the volume increase at a larger scale, causes an increase in internal resistance, empty volumes in which fermentation occurs, thus resulting in lower output compared to the smaller scale MFCs. To meet the requirement of high surface to volume ratio of the anodic half-cell, the macroporous and unmodified carbon veil could be used in increased geometry, which results in higher power as previously described [24]. However, this can be limited in the miniaturised designs to the available space of the anode chamber especially when multiple MFCs are assembled in modules. Further development will also require simple yet effective construction/maintenance procedures as finding low cost and scalable fabrication processes to achieve advanced porous carbon-based anode materials [42]. Optimisation of modified anode electrodes based on the loading of AC powder as well as based on various carbon-scaffolds (matrixes) will form part of future work. In MFC research, the vast majority of materials and modification strategies are unsuitable for practical applications due to scalability issues such as complex manufacturing processes and high costs, as well as their long-term stability, which is rarely reported or not given. This study is focused on the operational aspects of the power improvement in MFC technology and gives long-term operational data demonstrating stability of proposed system. The total cost of the carbon veil used for a single MFC was £0.31 and the cost of the activated carbon used for modification was £0.12 which gives the total of £0.43 making the fabrication method affordable.

### 3.5 Catholyte production

**Fig. 8:**
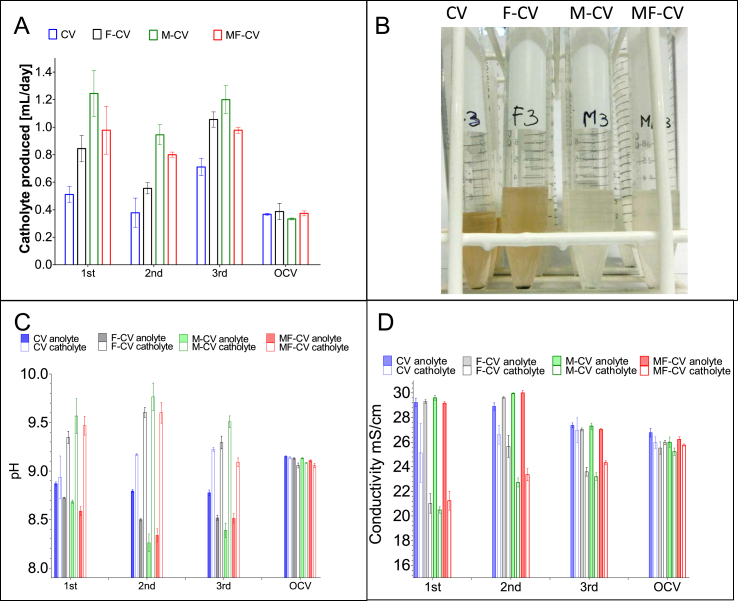
Catholyte volume recovered from all tested groups during closed circuit (three subsequent collections) and under open circuit (A), visual evidence of catholyte quality during second collection (B), pH splitting observed between anolyte and formed catholyte in batch fed MFCs during closed circuit (three subsequent collections) and under open circuit (C) and ion splitting measured as conductivity between the anolyte and formed catholyte in all MFC groups during closed circuit (three subsequent collections) and under open circuit (D).

Fig. 8A illustrates the volume of catholyte formed inside the ceramic reactors during the electricity generation. It shows that the largest volume of catholyte formed in group M-CV reaching up to 1.2 ± 0.2 mL day^−1^ and 1.0 ± 0.2 mL day^−1^ for MF-CV and F-CV, while the lowest production was at 0.7 ± 0.1 mL day^−1^ from CV type which also was the least powerful. It is worth noting that during the open circuit operation, the volume of catholyte was significantly lower and during the 2.5 months of OCV operation cathode flooding did not occur (Fig. 6B). Images of the catholyte collected during MFC operation illustrate that samples obtained from CV and F-CV were darker while the catholyte collected from MFCs with modified anodes were lighter in colour (close to transparent -Fig. 8B) which might suggest that both electroactive properties as well as adsorptive properties of activated carbon powder used for the modification could influence the permeate characteristics and influence purification. Permeate quality was assessed by the physicochemical properties including the pH (Fig. 8C), where the pH splitting was the highest under external load conditions and most significant in MF-CV (best performing) and lowest in CV (least performing) type. This is related to ORR activity and OH- production on the cathode elevating the pH according to the amount of produced charge. Ion splitting also occur where the catholyte under the MFC operation produces diluted (less concentrated) catholyte. The salt reduction can be noticeable when analysing the solution conductivity data in Fig. 8D, where the highest salt reduction, up to 30% in the catholyte comes from the modified anode (MF-CV and M-CV) MFCs which is related to power generation. This is in agreement with previous work which showed catholyte increase in pH and decrease in the conductivity from ceramic MFCs in comparison to urine based anolyte [39,43]. The significant decrease in ionic content of the catholyte filtrate is showing up to 30% reduction in comparison to anolyte (Fig. 8D) in the best performing MFCs which is due to the higher electrochemical activity [39] which in this case is determined by the anodic modification. Cation composition in hydrolysed urine is dominated primarily by ammonium ions reaching approximately 5 g L^−1^ but also Na^+^, K^+^, Ca^2+^ and Mg^2+^. NH^4+^ will be transported actively from the anode to the cathode as the proton carrier [44] whereas in the alkaline conditions, it will be transformed into volatile ammonia. With an appropriate ammonia stripping mechanism using sulphuric acid, the gaseous NH_3_ can then be transformed into a fertiliser [45]. As the catholyte is formed, the conductivity of the catholyte decreases (in comparison to the inflow urine and anolyte) due to the electroosmotic drag of water molecules accompanying the cations (primarily ammonium ion) transported to the cathode, dewatering the anolyte, as represented in Fig. 8C. This is similar to forward osmosis (FO), however the difference being that FO process is driven by the chemical potential [46], while here it is driven by the electrical force and the production of electric current resulting in partial desalination without an addition of draw solution or consumption of energy.

Future studies considering continuous modes of filtration with external catholyte collection as well as full chemical analysis would provide the basis for MFC system optimisation and performance. This would prevent cathode flooding and provide the separation and accumulation of the catholyte permeate. Improvements and further optimisation of the presented MFC system will result in a more sustainable biological treatment technology that has the potential for future scale-up in order to decrease energy requirement of treating waste.

## 4 Conclusions

The anodic surface of the carbon veil in microbial fuel cell can be functionalised with micro-nanostructure of the activated carbon particles to form low cost, interconnected and biocompatible carbonaceous network with large surface area that is suitable for the bacterial attachment. The modified anodes had slower start-up time due to the presence of the hydrophobic element, however they outperformed unmodified anodes after 10 days of operation. The maximum power performance from the modified MFCs operating on human urine reached up to 3.7 mW of power which is volumetric power density of 52.9 W m^−3^ and shows up to 3 times better performance to the control, unmodified anode. The production of electric current was the factor driving the electro-osmostic drag moving water through the ceramic membrane and resulting in constant production of purified catholyte with up to 30% salt reduction. These results show that simple and low cost anode modification by improving the surface characteristics of carbon veil scaffold can significantly enhance the power output and long term stability of the MFC reactor, mitigating cathode fouling.

## CRediT authorship contribution statement

**Iwona Gajda:** Conceptualization, Data curation, Formal analysis, Investigation, Methodology, Visualization, Writing - original draft, Writing - review & editing, Funding acquisition. **Jiseon You:** Methodology, Resources, Writing - review & editing. **Carlo Santoro:** Investigation, Resources, Writing - review & editing. **John Greenman:** Conceptualization, Supervision, Writing - review & editing. **Ioannis A. Ieropoulos:** Conceptualization, Funding acquisition, Project administration, Resources, Supervision, Writing - review & editing.

## Declaration of competing interest

The authors declare that they have no known competing financial interests or personal relationships that could have appeared to influence the work reported in this paper.

## Data Availability

The raw/processed data required to reproduce these findings cannot be shared at this time due to technical or time limitations.
